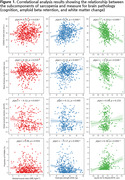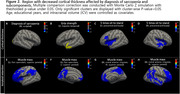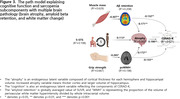# Identifying distinctive role of sarcopenia components for cognitive impairment with multimodal neuroimaging in non‐dementing older adults

**DOI:** 10.1002/alz.088584

**Published:** 2025-01-09

**Authors:** Sunghwan Kim, Sheng‐Min Wang, Dong Woo Kang, Yoo Hyun Um, Han Min Yoon, Soyoung Lee, Yeong Sim Choe, Regina EY Kim, Donghyeon Kim, Chang Uk Lee, Hyun Kook Lim

**Affiliations:** ^1^ Yeouido St. Mary's Hospital, College of Medicine, The Catholic University of Korea, Seoul, Korea Korea, Republic of (South); ^2^ Yeouido St. Mary's Hospital, Seoul, Seoul Korea, Republic of (South); ^3^ College of Medicine, Yeouido St. Mary's Hospital, The Catholic University of Korea, seoul, seoul Korea, Republic of (South); ^4^ College of Medicine, Seoul St. Mary's Hospital, The Catholic University of Korea, Seoul, Seoul Korea, Republic of (South); ^5^ St. Vincent' Hospital, College of Medicine, The Catholic University of Korea, Suwon, Gyeonggi‐do Korea, Republic of (South); ^6^ Brigham and Women's Hospital, Boston, MA USA; ^7^ Research Institute, Neurophet Inc., Seoul, Seoul Korea, Republic of (South); ^8^ Research Institute, Neurophet Inc., Seoul Korea, Republic of (South); ^9^ Seoul St. Mary's hospital, Catholic Medical College, the Catholic University of Korea, Seoul Korea, Republic of (South); ^10^ Medical College, the Catholic University of Korea, Seoul Korea, Republic of (South)

## Abstract

**Background:**

Although previous studies have demonstrated cognitive impairment in elderly individuals with sarcopenia and its neuronal substrates, there is no comprehensive model integrating multiple brain pathologies to predict cognitive impairment associated with sarcopenia. The aim of this study was to explore a comprehensive prediction model for cognitive impairment in sarcopenia using multimodal neuroimaging methods in non‐demented older adults.

**Method:**

This cross‐sectional study used data from the Catholic Aging Brain Imaging Database study, a population‐based cohort study with magnetic resonance imaging (MRI) scans, positron emission tomography (PET) scans, and clinical data. A total of 528 non‐demented older adults, with or without sarcopenia, was included. We measured three key components of sarcopenia: skeletal muscle index (SMI), hand grip strength (HGS), and the five‐times sit‐to‐stand test (5STS).

**Result:**

Sarcopenia measures related to muscle mass, power and speed demonstrated significant partial correlations with cognitive function. Cortical thickness was significantly associated with muscle power (HGS; left superior temporal cortex) and speed (5STS; bilateral precentral and insula cortex), while amyloid‐beta (Aß) hand distinctive association with muscle mass (SMI). In the path model, brain atrophy was primarily influenced by SMI and HGS through Aβ retention and periventricular white matter hyperintensity (pvWMH), respectively, resulting in cognitive impairment. On the other hand, the 5STS directly affected brain atrophy, contributing to cognitive decline.

**Conclusion:**

Sarcopenia sub‐domains, including muscle mass, power, and speed, showed distinctive associations with cognitive impairments related to Aß burden, pvWMH, and brain atrophy. Therefore, treatments targeting each sub‐domain should be considered to prevent cognitive decline associated with sarcopenia.